# Vascular Tumour at the Orifice of the Meatus Urinarius

**Published:** 1846-06

**Authors:** 


3. *Vascular Tumour at the Orifice of the Meatus Urinarius.*—A report of a case of this painful and troublesome affection, with some interesting remarks by Dr. Lever, are contained in the *London Medical Gazette*, (Jan. 9th, 1846.)

This disease was first described by Sir C. M. Clarke, (*Diseases of Females*, p. 289.) The patient whose case is related by Dr. Lever, was a woman 67 years of age, who suffered from pain in the urethra, irritability of the bladder, constant inclination to void its contents, obstruction in the passage of the urine, and a sense of scalding during its passage; pain in the pelvis, coursing to the back, hips, and thighs, was also complained of; she was weak, dyspeptic, dispirited, and worn out for want of sleep. Application to various persons for relief was vainly tried, until the true nature of the case was detected at the Chelsea Dispensary. The growth at the time of her admission was of a florid red color, and granulated; it protruded through the meatus urinarius, with the margin of which it was unconnected, but had rather a broad and short stalk attaching it with the canal at the distance of about ¼ of an inch. The slightest pressure caused the tumor to bleed, and the most gentle touch occasioned her exquisite pain. These growths appear to consist almost entirely of vessels and their connecting cellular tissue; they must, however, be abundantly supplied with nerves, from the exquisite suffering they occasion. Sometimes we meet with these growths in form and size like a small mulberry, having a slender stalk protruding through the meatus; sometimes these growths are no larger than a pea; in some cases they pass inwards along the urethra as far as, and even into the neck of the bladder, they seem to have their origin below the mucous membrane, and from the submucous cellular tissue. In forming a diagnosis of these tumors, we must not mistake for them simple polypoid tumors, which are occasionally found arising from within the urethra, and protruding through the meatus; it is true, they dam the flow of urine, the water passes in a small and stifled stream; there may be great efforts to empty the bladder, and if the tumour be long overlooked, the bladder may become thickened, and vesical irritation may ensue, but there is not that exquisite sensibility which is present where there are vascular growths, neither does the polypus so readily bleed. Again, malignant disease occasionally establishes itself at the orifice of the urethra, and this may exist without malignant disease of the uterus and vagina; here, also, there is difficulty in micturition, pain and scalding during the passage of the urine; there is occasional bleeding, and, conjoined with this, or in its absence, a mucous discharge. Occular examination will, however, readily detect the one from the other; in malignant disease there will be found a hardened lobulated tumour, or a cluster of lobulated tumours involving the urethra to a greater or less degree, diminishing its capacity and almost closing its external opening. And if, in addition to this, malignant disease of the vagina be present, it will greatly assist the diagnosis. Frequently, too, the inguinal glands are enlarged, and the aspect of the patient is characteristic of malignant cachexy. There is another disease to which this part of the female urinary apparatus is liable, viz.: thickening of the cellular membrane around the urethra, with an enlarged and varicose state of the vessels, in which there is a dilated state of the blood-vessels with an hypertrophied condition of the cellular membrane; the urethra for an inch or more behind the meatus is frequently so dilated as to hold some few drops of urine, which may be pressed from it, and which create continued irritation. This state of parts is accompanied by constant uneasiness; there may be pain in sexual intercourse, although for the most part females labouring under this malady have their sexual desires exalted; the uneasiness is increased in the erect posture, there is frequent inclination to evacuate the bladder both by night and by day, a small quantity of urine flowing at a time, and the patient generally feels as if there were more fluid to pass. There is also a slight mucous discharge.

The finger passed into the vagina feels the urethra to be swollen and spongy, and if the disease have lasted for some time, there will be a part trom which a few drops of urine may be pressed. When inspected, the part will be found of a dark red colour, and in some cases there is tenderness. Verrucous tumours growing from the vestibulum cannot readily be mistaken for this disease; the former are insensible, their colour resembles that of the part from which they grow, their number varies, they may be solitary, in other instancs there are many, but in all cases there is a mucous discharge.

"Let me advise you," says Dr. L., "in every case in which you are consulted, when the patient makes complaint of symptoms similar to those detailed in this woman's history, not to prescribe or give an opinion without the privilege of a tactile and visual examination. A neglect of these I have known lead the practitioner to commit sad mistakes, and involve himself in great disgrace. One instance I remember to have seen and treated, where the opinion given was, that there was calculus in the bladder. The patient as well as her friends was very properly alarmed; further advice was recommended; the case was investigated, no calculus was present, the sole disease being a vascular growth; this was removed, and the patient has had no return. I have on several occasions seen cases in which carcinoma uteri was suspected from the pain in micturition, the central pains attacking the pelvis and stretching to the back, hips, and down the thighs, and even this disease in some has been declared by the medical attendant to exist, although he had not availed himself of an internal or visual examination. Be on your guard, therefore; in no case give a hasty or rash opinion; take care not to judge by mere symptoms, without employing the several means of physical diagnosis which in a previous lecture I have detailed.

"Let me now request your attention to the treatment employed in this case; the neck of the tumour was grasped by a pair of forceps, and by means of a pair of scissors the mucous membrane of the urethra involved with the tumour itself was removed. To effect this, the patient should be held firmly, for, if she move, the structure of the growth is so slender that the tumour will tear away; the forceps employed should be broad, not the common artery forceps, for they will lacerate, and not hold firmly. The excision of these growths is frequently accompanied by a pretty copious bleeding; but it is rarely necessary to tie any vessel; a compress applied to the part for a time usually arrests the hemorrhage; this, however, should be looked to, especially if the patient call at your house, and has any distance to go after the operation; some arg. nit. was applied freely to the part from which the tumour was removed. After the slough separated, the wound looked healthy; there was no pain in passing the urine, which flowed in a full stream for some days. On the 7th, the granulations were sprouting: I then directed the clerk carefully to touch the part with arg. nit. dissolved in nit. acid; this I have found to be more potent, and I think less painful (if I may judge by the expressions of the patients,) than the arg. nit.; its effect has been good, and our patient will soon leave the hospital. In those cases where the tumour is of the form of a cherry or mulberry, I find the better plan is to tie a piece of dentist's silk waxed around the stalk, and snip off the tumour below; the silk should not be too thin, or it will cut through, neither should it be tied too tightly for the same reason. When the ligature comes away the stalk must be destroyed in the same way as in the case related; if the mucous membrane itself, and the submucous tissue, be not destroyed, the vascular tumour will most certainly reappear. The most troublesome forms of the complaint that we have to treat are those in which the tumour not only peeps through the meatus, but runs along the urethra, and in some instances passes into the cavity of the bladder; in such, the symptoms of irritation are intense, the stream of urine is as fine as a hair, and the suffering patient attempts to pass her water every three or five minutes. If the disease go on unrelieved, she wastes, becomes dispirited, dyspeptic, and may at last die, worn out by her long-continued and aggravated sufferings. In such cases we cannot remove by scissors or knife the vascular growth within the urethra; the first thing to be done, therefore, is to pass a small sound or catheter to establish a canal for the passage of the urine. However painful this must be, and agonizing it most certainly is, it must be done; some arg. nit. must then be passed along the track of the urethra, and by its agency the vascular growth must be destroyed; the sound or catheter must be passed every day or every second day according to circumstances, and the arg. nit. repeated as soon as the slough occasioned by its use has separated; it is as well to let the patient keep the sound or bougie in the bladder for half an hour after the application of the caustic.

"This is certainly the most difficult form of the tumour to treat, and unless we succeed in effectually destroying the structure from which the tumour proceeds, we shall most certainly have it reappear. While this treatment is had recourse to, the patient must be closely watched, for I have seen cystitis occasioned more than once by the caustic applied in the manner I have recommended; but this will depend on the constitution and susceptibility of the patient, as well as upon the state of the mucous membrane of the bladder itself.

"Various other modes of treatment have been recommended—the application of the Tr. Iodinæ c. Pulv. Sabinæ, Pulv. Alum, &c. All I have tried, with some I have occasionally succeeded, with all I have many times failed; the plan I adopted in this woman's case is the one I believe to be in the majority of cases the most successful.

"When the cause of the continued irritation was removed, this patient's health and spirits quickly recovered, and at the present time she looks remarkably well and lively for a woman of 64."—*Am. Jour. of Med. Sci.*, for April.

